# High risk for latent tuberculosis infection among medical residents and nursing students in India

**DOI:** 10.1371/journal.pone.0219131

**Published:** 2019-07-08

**Authors:** Aarti Kinikar, Ajay Chandanwale, Dileep Kadam, Samir Joshi, Anita Basavaraj, Geeta Pardeshi, Sunita Girish, Sangeeta Shelke, Andrea DeLuca, Gauri Dhumal, Jonathan Golub, Nilima Lokhande, Nikhil Gupte, Amita Gupta, Robert Bollinger, Vidya Mave

**Affiliations:** 1 Department of Pediatrics, BJGMC-JHU Clinical Trial Unit, Byramjee-Jeejeebhoy Government Medical College, Pune, Maharashtra, India; 2 Department of Orthopedics, BJGMC-JHU Clinical Trial Unit, Byramjee-Jeejeebhoy Government Medical College, Pune, Maharashtra, India; 3 Department of Medicine, Smt. Kashibai Navale Medical College and General Hospital, Pune, Maharashtra, India; 4 Department of ENT, BJGMC-JHU Clinical Trial Unit, Byramjee-Jeejeebhoy Government Medical College, Pune, Maharashtra, India; 5 Department of Medicine, Government Medical College, Miraj, Maharashtra, India; 6 Department of Community Medicine, Vardhman Mahavir Medical College and Safdarjung Hospital, New Delhi, India; 7 Department of Biochemistry, BJGMC-JHU Clinical Trial Unit, Byramjee-Jeejeebhoy Government Medical College, Pune, Maharashtra, India; 8 Department of Community Medicine, BJGMC-JHU Clinical Trial Unit, Byramjee-Jeejeebhoy Government Medical College, Pune, Maharashtra, India; 9 Department of Environmental Health and Engineering, Bloomberg School of Public Health, Johns Hopkins University, Baltimore, MD, United States of America; 10 BJGMC-JHU Clinical Trial Unit, Byramjee-Jeejeebhoy Government Medical College, Pune, Maharashtra, India; 11 Department of Medicine, Epidemiology and International Health, Johns Hopkins University School of Medicine and the Johns Hopkins Bloomberg School of Public Health, Baltimore, Maryland, United States of America; 12 Department of Medicine, Division of Infectious Diseases, Johns Hopkins University School of Medicine, Baltimore, Maryland, United States of America; University of Washington, UNITED STATES

## Abstract

Defining occupational latent tuberculosis infection (LTBI) risk among healthcare workers is needed to support implementation of prevention guidelines. Prospective cohort study of 200 medical residents and nursing students in India was conducted May 2016—December 2017. Tuberculin skin test (TST) and QuantiFERON TB Gold Test-in-tube (QFT-GIT) were performed at study entry and 12 months. Primary outcome was incident LTBI (≥10mm TST induration and/or ≥0.35IU/mL QFT-GIT) at 12 months; secondary outcomes included baseline LTBI prevalence and risk factors for incident and prevalent LTBI using Poisson regression. Among 200, [90 nursing students and 110 medical residents], LTBI prevalence was 30% (95% CI, 24–37); LTBI incidence was 26.8 (95% CI, 18.6–37.2) cases per 100 person-years and differed by testing method (28.7 [95% CI, 20.6–38.9] vs 17.4 [95% CI, 11.5–25.4] cases per 100 person-years using TST and QFT-GIT, respectively). Medical residents had two-fold greater risk of incident LTBI than nursing students (Relative Risk, 2.16; 95% CI, 1.05–4.42). During study period 6 (3%) HCWs were diagnosed with active TB disease. Overall, median number of self-reported TB exposures was 5 (Interquartile Range, 1–15). Of 60 participants with prevalent and incident LTBI who were offered free isoniazid preventive therapy (IPT), only 2 participants initiated and completed IPT. High risk for LTBI was noted among medical residents compared to nursing students. Self-reported TB exposure is underreported, and uptake of LTBI prevention therapy remains low. New approaches are needed to identify HCWs at highest risk for LTBI.

## Introduction

Tuberculosis (TB) risk is elevated among health care workers (HCW) in low- and middle-income countries (LMIC) compared to the general population [1–3]. While global estimates report a range of incident TB disease among HCWs (69–5780 cases per 100 000) [1], our group has estimated more than 8-fold higher TB disease incidence (1788 cases per 100 000 HCW-years) among HCWs compared to community members (212 cases per 100 000 person-years) in the same region [4]. In addition, we have reported particularly high TB disease risk among new medical trainees at 3279 cases per 100 000 person-years [5]. Although occupational TB exposure has gained increased attention and awareness, latent TB infection (LTBI), an indicator of TB exposure, remains poorly characterized in high TB burden settings.

Globally, average LTBI prevalence among HCWs is estimated at 54% with annual risk ranging from 0.5% to 14% [6–10]. Further, medical residents have been found to be at higher risk for LTBI than other students in LMIC, indicating high TB disease exposure in the workplace [11–13]. Low awareness of and control over exposure to risk factors likely contribute to this risk, yet risk factors for LTBI are not well-defined, particularly among healthcare workers in high TB burden countries. Further, although World Health Organization (WHO) guidelines are in place for infection control in LMIC [14, 15], precise LTBI estimates are unknown.

As recommended by WHO, most national programs in LMIC use a tuberculin skin test (TST) to calculate prevalence and incidence, and research programs use an interferon gamma release assay (IGRA) to assess LTBI [16], yet neither method is considered a gold standard for measuring LTBI. We, therefore, undertook a prospective study to estimate risk of LTBI using TST and IGRA and examine risk factors for LTBI among a cohort of medical residents and nursing students employed in a public-sector teaching hospital in western India.

## Methods

### Study population and design

A prospective longitudinal cohort of health care workers (HCWs) comprising post graduate medical residents and nursing students pursuing a bachelor’s degree in nursing was established at Byramjee-Jeejeebhoy Government Medical College (BJGMC) and Sassoon General Hospital (SGH) in Pune, Maharashtra, between May 2016 and December 2017. BJGMC-SGH is a public sector, tertiary care, teaching hospital serving the surrounding urban, semi-urban and rural population of an estimated seven million people. Consenting HCWs participated in the study. HCWs aged <18 years or with prior history of TB disease were excluded.

### Study procedures

All participants provided written informed consent. At study entry: socio-demographic and clinical characteristics were assessed, including comorbid illnesses, current self-reported TB exposure and prior TB disease exposure in the community as well as in the clinical settings, Bacille Calmette-Guerin (BCG) vaccination, and; a WHO-recommended TB symptom questionnaire was administered; and physical examination was conducted, including anthropometric measurements and visualization of BCG scar. IGRA testing using QuantiFERON TB Gold Test-in-tube (QFT-GIT, Cellestis) and TST were performed at study entry and at 12 months. Additional IGRA testing was performed at months 1, 3, 6 and 9. Self-reported TB exposures for the previous year both in the wards/clinics and in the community were collected at the end of the study [17].

QFT-GIT testing was performed and analyzed according to manufacturer instructions (QIAGEN, Germany; QFT-G analysis software -Version: 2.62). Three milliliters of blood was collected for QFT-GIT testing; blood was collected prior to placement of TST. Using an enzyme-linked immunosorbent assay reader, interferon gamma (IFN-ɣ) levels (IU/ml) were estimated (ELx808, BioTek, USA) and considered positive if the value of the TB Antigen minus Nil control was ≥0.35 IU/ml and >25% of Nil value. TST (Span Diagnostics, India) was administered by the study staff; 0.1 ml (5 units) of Purified Protein Derivative (PPD) was placed intradermally on the flexor aspect of the forearm. The reaction was read 48–72 hours later. The size of the reaction was determined by measuring the transverse diameter of the induration.

Sputum acid-fast bacilli (AFB) smear, Gene Xpert and culture were obtained from any participant with symptoms or clinical presentation concerning for active TB. Participants with suspected extra-pulmonary TB (EPTB) underwent additional AFB smear, culture and histopathological evaluation of the affected sites if needed. AFB cultures were performed on Lowenstein Jenson media (solid culture) and Mycobacterial Growth Indicator Tube (MGIT, liquid culture). The BJGMC-SGH Institutional Ethics Committee and Johns Hopkins University Institutional Review Board approved all study methods to conduct the study at SGH.

### Primary and secondary outcomes

The primary study outcome was an estimate of LTBI incidence calculated as the number of participants without prevalent LTBI (positive TST [≥10mm TST induration] and positive QFT-GIT [OD≥0.35IU/mL] and active TB at study entry who subsequently developed incident LTBI (positive TST [≥10mm TST induration] and/ or positive QFT-GIT [OD≥0.35IU/mL] at month 12 divided by the total duration of TB exposure multiplied by 100. Secondary outcomes included prevalent LTBI, risk factors for both incident and prevalent LTBI, LTBI incidence by TST vs. QFT-GIT, and time to incident LTBI by serial QFT-GIT testing, prevalent and incident TB disease. Prevalent LTBI was calculated as the number of participants with baseline LTBI divided by the total number of study participants tested. Individual, exposure-related variables were assessed as risk factors for incident and prevalent LTBI. Prevalent TB disease was defined as a diagnosed TB disease up to 3 months after study entry.

### Statistical analyses

Baseline categorical and continuous variables for socio-demographic and clinical characteristics were summarized using proportions and medians with interquartile range (IQR), respectively, and compared; P-values ≤0.05 were deemed statistically significant. Prevalent LTBI was calculated as a proportion with corresponding 95% exact binomial CI for several exposures of interest. Similarly, LTBI incidence rates were calculated per 100 person-years for several exposures of interest with Poisson exact 95% confidence intervals. To explore risk factors for incident and prevalent LTBI, univariable or multivariable logistic and Poisson regression was performed as appropriate. Time to LTBI by IGRA was estimated using Kaplan-Meier methods and compared among medical and nursing trainees using a log-rank test. Data were analyzed using STATA (version 13.1).

## Results

### Study population

Of 200 participants, 110 (55%) were medical residents, 90 (45%) were nursing students, median age was 25 years (IQR, 19–27), and 113 (56%) were female. At study entry, 47 (24%) had a body mass index >25 kg/m^2^, 30 (15%) reported alcohol use, 20 (10%) reported smoking tobacco, 89 (45%) reported prior exposure to known sputum smear positive TB, and 38 (19%) reported exposure to a person with active TB disease outside the healthcare setting (**S1 Table**).

### Prevalent latent tuberculosis infection

**Fig 1:**
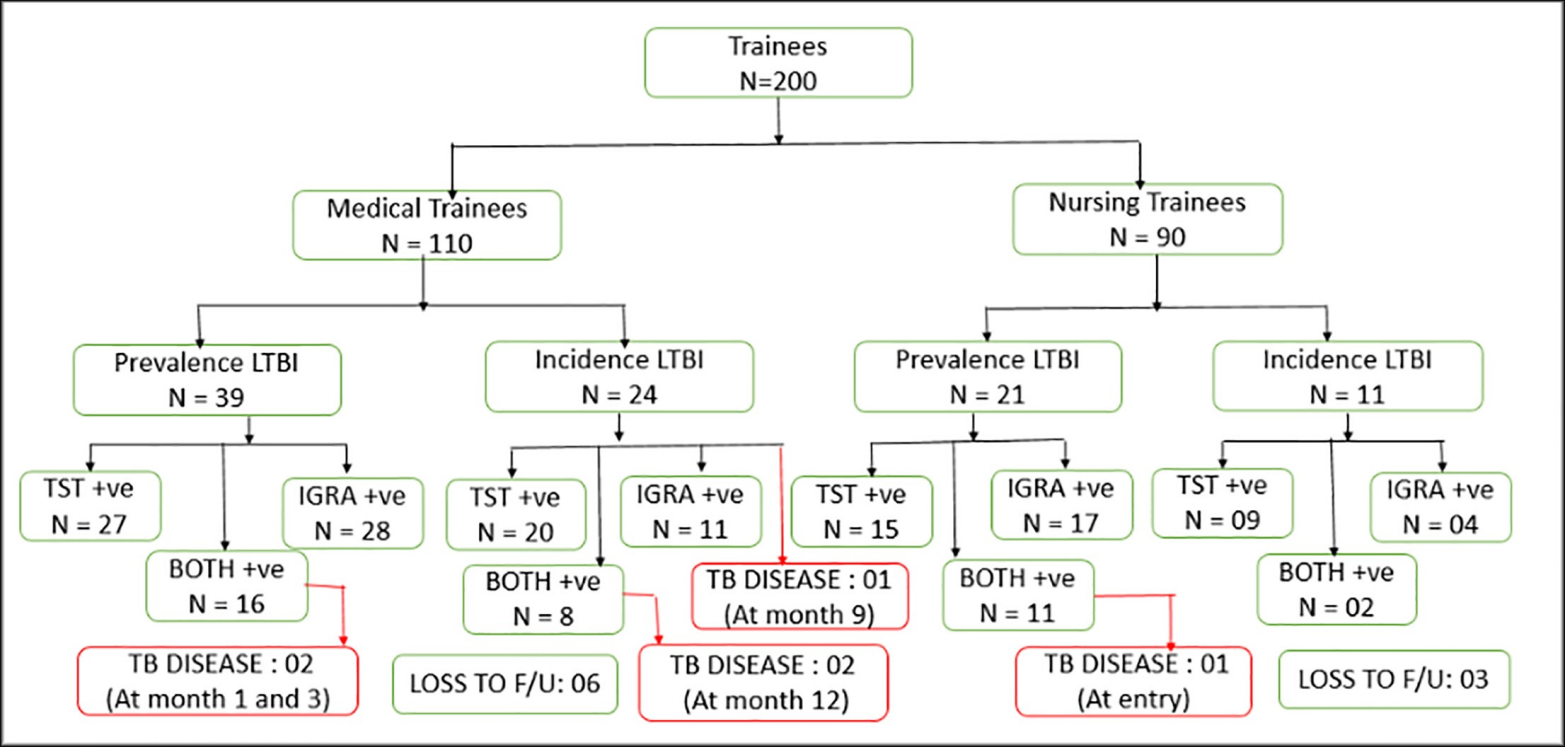
Flow diagram showing prevalent and incident latent tuberculosis infection by trainee type and testing method. Latent tuberculosis infection (LTBI) was assessed among medical residents and nursing students at study entry and 12 months using tuberculin skin test (TST) and interferon gamma release assay (IGRA).

At study entry, LTBI prevalence was 30% (95% CI, 24–37%) overall and higher among medical residents than nursing students, although not statistically significant (35% [95% CI, 27–45] vs. 23% [95% CI, 15–33], p = 0.06) (**S1 Table** and **Fig 1**). LTBI prevalence was seen in 42 (21%) by TST, 45 (22.5%) by QFT-GIT and 27 (13.5%) by both. LTBI prevalence by TST was 30 (22%) in those with BCG scar while 30 (22%) with QFT-GIT. Median TST at baseline was 0 (IQR 0–7). Prior exposure to sputum smear positive TB cases was associated with two-fold greater risk of prevalent LTBI than no history of TB exposure (Relative Risk [RR], 2.11; 95% CI, 1.04–4.24). Notably, three (1.5%) participants had active TB disease, one nursing student at baseline, two medical resident during follow up at month 1 and 3, respectively; all three had both positive TST and QFT-GIT at baseline.

### Incident latent tuberculosis infection

**Fig 2:**
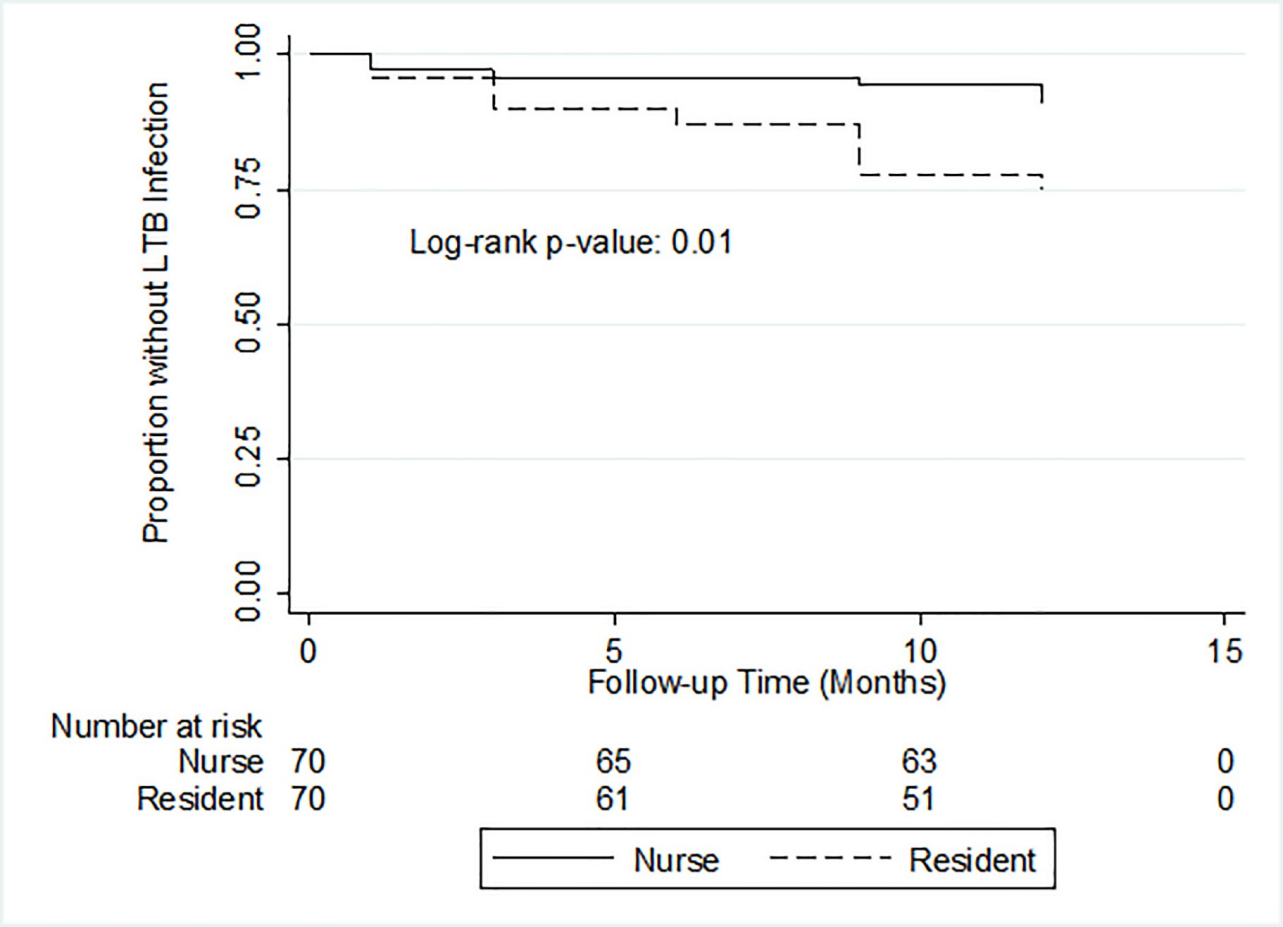
Kaplan Meier curve showing time to incident latent tuberculosis infection by trainee type. Incident latent tuberculosis infection was identified using serial QuantiFERON TB Gold Test-in-tube performed at 1, 3, 6, 9, and 12 months among medical residents and nursing students without baseline latent tuberculosis infection (n = 131).

**Fig 3:**
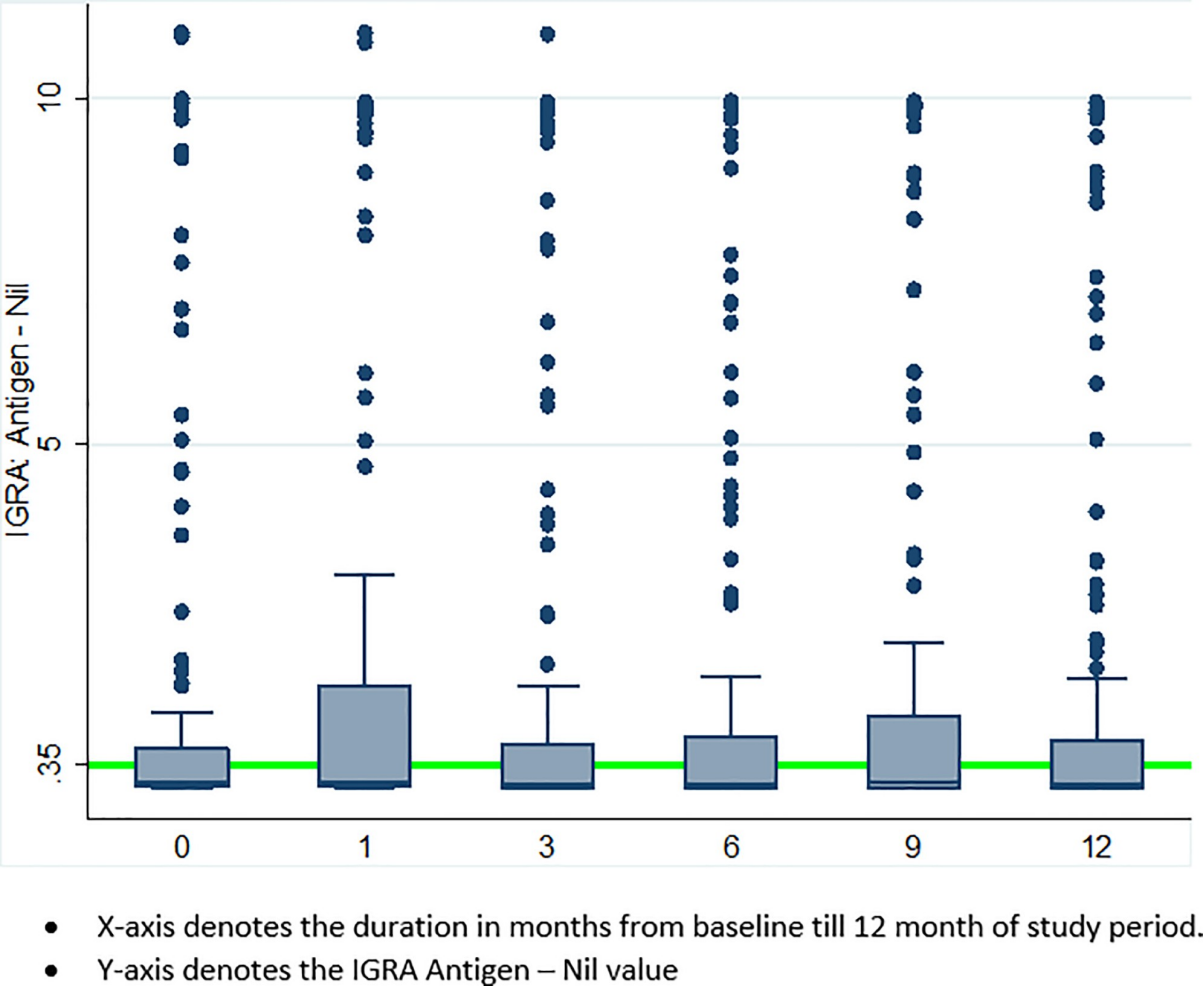
Box-plot figure showing the longitudinal QFT-GIT values among trainee health care workers. (A) X-axis denotes the duration in months from baseline till 12 months of study period. (B) Y-axis denotes the IGRA Antigen–Nil value. This figure depicted the longitudinal QFT-GIT values among trainee health care workers (**Fig 3**).

**Fig 4:**
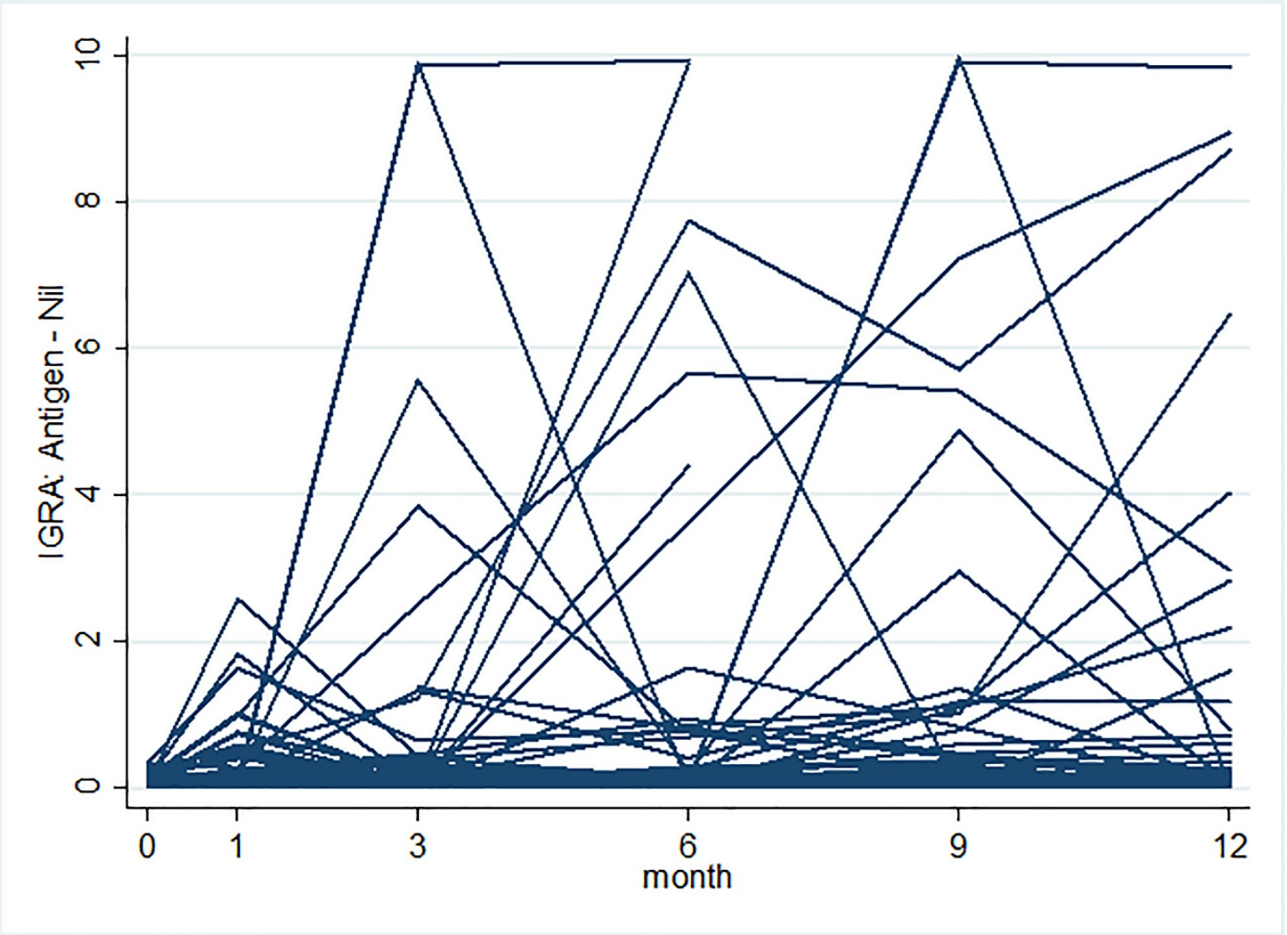
IGRA results over time among those with no LTBI at baseline. It depicts individual participants QFT-GIT profile who were negative LTBI at baseline and who underwent serial QFT-GIT testing at various time point (month 1, 3, 6, 9 and 12) upto 12 month (**Fig 4**).

Of 140 trainees without prevalent LTBI, nine were lost to follow up and excluded from the analysis. Of the 131 remaining trainees, one medical resident developed active TB disease at month 9, and 35 (27%) trainees tested positive for LTBI at month 12, including two medical residents diagnosed with active TB disease (**Fig 1**). Overall, the estimated LTBI incidence at 12 months was 26.8 cases per 100-person years (95% CI, 18.6–37.2) (**Table 1**) and differed by testing method. Of 143 trainees with negative baseline TST, 43 were TST-positive at month 12, contributing to an estimated LTBI incidence rate of 28.7 cases per 100-person years (95% CI, 20.6–38.9) using TST; with median TST induration among those with negative TST at baseline was 0 (IQR 0–14); of 155 trainees with negative baseline QFT-GIT, 26 seroconverted at month 12, contributing to an estimated LTBI incidence rate of 17.4 cases per 100-person years (95% CI, 11.5–25.4) using QFT-GIT (**Fig 1**). The median time to incident LTBI (IQR) using serial QFT-GIT was 6 (3–9) months overall and significantly shorter among medical residents compared to nursing students (3 months vs. 6 months, p = 0.01) (**Fig 2**). Furthermore, as shown in Fig 3, QFT-GIT values converted to negative during one year follow-up. However Fig 4, highlighted the QFT-GIT values of individual participants who had no LTBI at baseline.

**Table 1:** Rate of incident latent tuberculosis infection and risk factors among healthcare trainees in Pune, India (n = 131).

	Overall	Incident LTBI		Relative Risk
Risk Factor	N	N (%)	95% CI	RR (95% CI)
**Gender**				
**Male**	60	18 (30.1)	17.8–47.6	1
**Female**	71	17 (23.9)	13.9–38.3	0.79 (0.41–1.54)
**Median age, y(IQR)**	21 (19–26)	25 (20–27)		
**Increasing age by 1 y**				**1.08 (1.02–1.14)**
**BMI, kg/m^2^**				
**18.5–25**	81	22 (27.2)	17.1–41.2	1
**<18.5**	26	5 (19.2)	6.2–44.9	0.71 (0.27–1.87)
**>25**	24	8 (33.3)	14.4–65.7	1.22 (0.55–2.75)
**Tobacco smoking**				
**No**	115	29 (25.2)	16.9–36.2	1
**Yes**	15	6 (40.5)	14.9–88.2	1.61 (0.67–3.87)
**Alcohol use**				
**No**	108	26 (24.1)	15.7–35.3	1
**Yes**	23	9 (39.5)	18.5–74.9	1.64 (0.77–3.50)
**Trainee type**				
**Nursing student**	66	11 (16.7)	8.3–29.8	1
**Medical resident**	65	24 (37.0)	23.7–55.1	**2.22 (1.09–4.53)**
**Exposure to PTB case^a^**				
**No**	25	6 (24.0)	8.8–52.2	1
**Yes**	101	27 (26.8)	17.7–39.0	1.12 (0.46–2.70)
**Median number of TB case exposures^a^, N(IQR)**	5 (1–15)	4 (1–33)		
**Number of TB case exposures^a^**				
**<15**	82	18 (22.0)	13.0–34.7	1
**>15**	18	6 (33.3)	12.2–72.6	1.51 (0.60–3.83)
**BCG scar**				
**No**	39	11 (28.2)	14.2–50.7	1
**Yes**	90	24 (26.7)	17.1–39.0	0.94 (0.46–1.92)
**Unknown**	2	0	0	-

BCG, Bacille Calmette-Guerin; BMI, body mass index; CI, confidence interval; IQR, interquartile range; LTBI, latent tuberculosis infection; PTB, pulmonary tuberculosis; RR, relative risk; TB, tuberculosis.

^a^Over the past one year.

In regression analysis (**Table 1**), increasing age (RR, 1.08; 95% CI, 1.02–1.14), and being a medical resident (RR, 2.22; 95% CI, 1.09–4.53) were associated with increased risk for incident LTBI. The median number of self-reported, workplace TB exposures over 12 months was 5 (IQR, 1–15), and although not statistically significant, participants reporting >15 TB case exposures in one year had >50% higher risk for incident LTBI (RR, 1.51; 95% CI, 0.60–3.83). Estimated LTBI incidence among the 18 (14%) participants reporting ≥15 TB case exposures over 12 months was 33.3 (95% CI, 12.2–72.6) cases per 100-person years compared to 22.0 (13.0–34.7) cases per 100-person years among those reporting <15 TB exposures. Notably, only three participants reported consistent use of a N95 mask (>95%) in wards and clinics during working hours. All participants with prevalent and incident LTBI were offered isoniazid preventive therapy (IPT) as required by the study protocol, yet only two (one medical resident and one nursing student) agreed to IPT and completed the 6-month course. The main reasons for IPT refusals reported by HCWs were–it is not recommended by Revised National Tuberculosis Control Program (RNTCP) [24, (35.3%)], prolonged duration of IPT treatment [19, (28%)] and concern about development of TB resistant to INH [14, (20.6%)].

## Discussion

Our prospective cohort study of prevalent and incident LTBI among medical residents and nursing students in India has several important findings. Approximately 30% of them had prevalent LTBI at study entry using either TST or IGRA. Risk of incident TB infection at 12 months was 27% among them. Our analysis found that TST detected 1.6-fold greater LTBI incidence compared to IGRA. Overestimation of incidence of LTBI due to TST may be because of boosting effect of baseline TST. TST boosting generally occurs within 5 weeks of previous negative TST and repeat TST in our study was performed at 12 months which was outside of this 5-week window [18, 19]. Furthermore, notably, history of exposure to sputum positive TB patients significantly increased the risk of prevalent LTBI. Being a medical resident and increasing age were associated with higher risk of incident LTBI, suggesting that greater TB exposure increases risk even though our analysis found no significant association between any exposure variable (i.e. any TB exposure, pulmonary TB exposure, or >15 TB exposures) and incident LTBI. However, self-reported TB exposure and uptake of TB prevention measures were strikingly low in our study.

In our high TB burden, LMIC setting, the risk of incident LTBI over 12 months (27%) is nearly two-fold higher than the highest range of estimated annual TB infection risk (0.5–14%) reported for HCWs globally [2, 3]. We have previously reported that medical residents have 15-fold higher risk of acquiring TB disease than the general population [5]. We have also reported that medical residents and nursing students are at risk for multi-drug resistant (MDR) TB. Therefore, it is unsurprising that rates of prevalent and incident LTBI are also high among healthcare workers.

Our analysis indicates that medical residents are at two-fold higher risk of acquiring LTBI than nursing students, and time to incident LTBI was significantly shorter for medical residents. Such increased risk is expected given that medical residents have more direct interactions with patients in wards and clinics than nursing students in our setting. Prior studies have also reported that time spent in the wards and clinics is an independent risk factor for TB [20, 21]. Similarly, we identified that increasing age was associated with higher LTBI risk. This is because medical residents are older than nursing students, and as mentioned before, medical residents have higher exposure. Also, the residents have spent more time in the hospital during their MBBS training, and internship. Since the exposure period is longer, residents are at a higher risk of incident TBI.

Another significant finding of our study is the low TB exposure reported among trainees. The median number of self-reported TB exposures was only 5 over a one-year period, yet approximately 2500 patients are diagnosed with TB in our hospital each year. The discrepancy suggests that HCWs may underestimate their TB exposure in health care settings. Importantly, perception of low occupational TB exposure could be a driving factor for lower uptake of WHO AIC guidelines as reported by our prior study [22, 23].

Efforts to prevent TB exposure and progression to active disease are critical in this high risk population. In the present study, we observed that few healthcare trainees used personal protective equipment (PPE), and participants were unwilling to start IPT even when freely available. Further, HCWs with incident and prevalent LTBI become a reservoir at risk for developing active TB disease, including MDR-TB, in Indian health care settings. Previous reports have demonstrated that newly infected nurses are at 10-fold higher risk for developing active TB and TB-related mortality than those with prevalent LTBI, and a recent meta-analysis attributed over 81% of active TB cases among HCWs to occupational exposure in high TB burden countries [24, 25]. Such studies provide evidence that IPT may have a critical role among those with incident LTBI. However, as seen in our study population, IPT uptake and completion remain low, suggesting the need for improved education, easy access and enhanced counselling. It is critical to improve the infection control practices in high TB burden countries, such as India, to interrupt TB transmission [26].

Our study has several limitations. First, our sample size is small, but our study included both medical residents and nursing students, offering a snapshot of differential LTBI risk in Indian healthcare settings. Next, because exposure to TB cases was self-reported and collected at the end of the study, this variable may be less reliable due to recall bias. Notably, some experts believe that BCG vaccination may be a reason for TST reactivity, however the effect of BCG vaccination wanes over time and most experts believe that TST positivity is likely due to new infection rather than BCG [18]. While prior studies have reported on the unreliability of self-reporting short contacts, another major challenge in assessing exposure in health care settings would be exposure to unknown or undiagnosed TB cases [27]. A better measure to assess the association of number of exposures to risk of LTBI would be to obtain real time data on exposure rather than collect annual data as recommended by the CDC, which is subject to recall bias. Finally, we did not collect information on time spent in the wards and clinics or number of encounters with active TB patients, thus potentially underestimating the association of exposure with incident LTBI.

In conclusion, our study highlights the increased risk of LTBI among HCWs in high TB burden countries and identifies medical residents to be at highest risk of contracting LTBI. While underuse of PPE is overwhelming, lower perceived risk as indicated by low self-reported exposure underscores the urgent need for education and improving the awareness of personal TB infection risk among HCWs. Urgent measures and strategies are needed to implement the long-recommended WHO AIC measures, including consistent use of PPE to mitigate risk of occupational TB in LMIC health care settings.

## Supporting information

S1 Table Rate of prevalent latent tuberculosis infection and risk factors among healthcare trainees in Pune, India (n = 200). CI, confidence interval; IQR, interquartile range; LTBI, latent tuberculosis infection; PTB, pulmonary tuberculosis; TB, tuberculosis.
(DOCX)
