# Supplementary material for: High risk for latent tuberculosis infection among medical residents and nursing students in India
Source: PLoS One. 2019 Jul 8;14(7):e0219131. doi: 10.1371/journal.pone.0219131 (PMC6613683; doi:10.1371/journal.pone.0219131)
Supplement: S1 Table — CI, confidence interval; IQR, interquartile range; LTBI, latent tuberculosis infection; PTB, pulmonary tuberculosis; TB, tuberculosis. (DOCX) [file pone.0219131.s001.docx]

| **S1 Table. Rate of prevalent latent tuberculosis infection and risk factors among healthcare trainees in Pune, India (n=200)** | | | | |
| --- | --- | --- | --- | --- |
| **Risk Factor** | **Overall, N (%)** | **Prevalent LTBI, N (%) [95% CI]** | ***P*** | **Relative Risk [95% CI]** |
| Gender  Male  Female | 87 (44)  113 (56) | 25 (29) [19 - 38]  35 (31) [22 - 40] | 0.73 | 1  1.11 [0.60 – 2.05] |
| Median age, y (IQR) | 25 (19 – 27) | 25 (20 – 28) | **0.01** | 1.09 [1.02 – 1.16] |
| Body mass index, kg/m^2^  18.5-25  <18.5  >25 | 115 (58)  38 (19)  47 (24) | 28 (24) [16 - 32]  11 (29) [14 - 44]  21 (45) [30 - 59] | Ref  0.57  **0.01** | 1  1.27 (0.56 – 2.87)  **2.51 (1.23 – 5.13)** |
| Tobacco smoking  No  Yes | 178 (90)  20 (10) | 54 (30) [24 - 38]  5 (25) [9 - 49] | 0.62 | 1  0.77 (0.26 – 2.21) |
| Alcohol use  No  Yes | 170 (85%)  30 (15%) | 53 (31) [24 - 39]  7 (23) [10 - 42] | 0.39 | 1  0.67 (0.27 – 1.66) |
| Trainee type  Nursing student  Medical resident | 90 (45%)  110 (55%) | 21 (23) [15 - 33]  39 (35) [27 - 45] | 0.06 | 1  1.80 (0.97 – 3.37) |
| Sputum positive PTB exposure  No  Yes  Not sure | 76 (38%)  89 (45%)  35 (18%) | 16 (21) [13 - 32]  32 (36) [26 - 47]  12 (34) [19 - 52] | Ref  **0.04**  0.14 | 1  **2.11 (1.04 – 4.24)**  1.96 (0.80 – 4.76) |
| Community TB exposure  No  Yes  Not sure | 142 (71%)  38 (19%)  20 (10%) | 38 (27) [20 - 35]  15 (39) [24 - 57]  7 (35) [15 - 59] | Ref  0.13  0.44 | 1  1.78 (0.84 – 3.78)  1.47 (0.55 – 3.97) |
| Hospital TB exposure  No  Yes  Not sure | 136 (68%)  37 (19%)  27 (14%) | 37 (27) [20 - 35]  13 (35) [20 - 53]  10 (37) [19 - 58] | Ref  0.35  0.30 | 1  1.45 (0.67 – 3.14)  1.57 (0.66 – 3.75) |
| Any TB exposure  No  Yes  Not Sure | 69 (35%)  101 (51%)  30 (15%) | 13 (19) [10 - 30]  37 (37) [27 - 47]  10 (33) [17 - 53] | Ref  0.27  0.12 | 1  2.49 (1.20 – 5.15)  2.15 (0.82 – 5.68) |
